# Role of Phospholipids on Drug Dissolution in Polymer Solid Dispersions Prepared by Hot-Melt Extrusion

**DOI:** 10.1021/acsomega.5c01861

**Published:** 2025-07-15

**Authors:** Danilo Monteiro de Carvalho, Ana Carolina Mendes Lourenço, Guilherme Gomes Moreira, Fritz Eduardo Kasbaum, Ana Luiza Lima, Marcilio Cunha-Filho, Stephânia Fleury Taveira, Ricardo Neves Marreto

**Affiliations:** † Laboratory of Nanosystems and Drug Delivery Devices (NanoSYS), School of Pharmacy, Universidade Federal de Goiás (UFG), 74605170 Goiânia, GO, Brazil; ‡ Laboratory of Food, Drug, and Cosmetics (LTMAC), School of Health Sciences, 505616 Universidade de Brasilia (UnB), 74910900 Brasília, DF, Brazil

## Abstract

The development of solid dispersions (SDs) has gained increased attention in recent decades, leading to successful delivery systems for various marketed products. In general, combinations of polymers and lipids in SD formulations have shown promising results in reducing the disadvantages associated with the isolated use of hydrophilic excipients such as copovidone (COP) and Soluplus (SLP). This study investigated the influence of the phospholipid (soy phosphatidylcholine, PPC, 15 and 30%, w/w) on the dissolution performance of drug-polymer SDs prepared by hot melt extrusion. A low-solubility model drug (ritonavir, RTV) was selected for the study. A complete drug amorphization was observed for all extrudates despite the PPC presence. However, PPC improved the process yield without requiring a plasticizer. Morphology and particle size analyses revealed the effects of PPC addition in the aqueous dispersions prepared from SDs, denoting a higher polymer–lipid interaction in COP dispersions and the formation of micrometric structures in both COP and SLP dispersions. COP-based SDs produced almost instantaneous increases in RTV dissolution of up to 7-fold, while SLP-based SDs achieved progressive increases over 5-fold. Importantly, PPC incorporation in COP-based SDs did not have an apparent effect on RTV dissolution but significantly improved drug dissolution from the SLP-based SDs. In summary, the role of the lipid mainly depends on the drug-polymer interactions and lipid concentration. Adding phospholipids enables the thermal process without needing other adjuvants.

## Graphical abstract

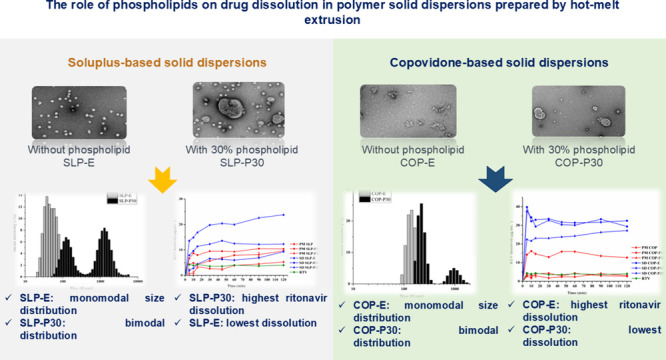

## 1 Introduction

Poorly water-soluble drugs are one of the most significant hurdles in the development of pharmaceutical formulations due to their low oral absorption and bioavailability. Pharmaceutical industries and the scientific community have been keen on exploring technological strategies to develop formulations with enhanced dissolution rates and absorption, thereby improving their therapeutic effectiveness.^1^

In this scenario, solid dispersions (SDs) have gained attention in the past decades, leading to promising delivery systems for various marketed products.^1–3^ This approach can improve biopharmaceutical properties through particle size reduction, drug dispersion at the molecular level, and increased wettability. These mechanisms promote the drug’s supersaturation state in biological fluids, thus accounting for a higher passive absorption by intestinal cells.^3,4^

The physicochemical properties of excipients used in the SD formulations and their specific intermolecular interactions are essential in achieving and maintaining supersaturation states in a wide range of pH.^5,6^ In particular, combinations of polymers and lipids have shown promising results in reducing the disadvantages of the isolated application of hydrophilic excipients, ensuring optimal stability and performance.^7,8^ Nevertheless, the enhancement of dissolution rates depends on the nature of the lipid-polymer interaction, which may be influenced by the combination of materials.^8^

Further to the effects of excipient association, the technique used in SD preparation can affect the properties of the final drug product. Various studies have demonstrated extensive differences in dissolution rates of physical mixtures and hot melt extrudates in aqueous medium.^6,8–11^ Hot melt extrusion (HME) is a solvent-free process highly efficient in obtaining amorphous SDs due to its capacity to yield molecular-level mixtures.^10,12,13^ Despite the limited number of studies on lipid-polymer SDs using HME, the results are encouraging, with high dissolution rates,^14^ indicating that this approach requires more investigation.

In this study, six SDs based on mixtures of copolymers Soluplus and Plasdone S-630 (copovidone) in association with Lipoid S100 (amphiphilic lipid soybean phosphatidylcholine) were processed by HME. The effect of each polymer–lipid combination on the physicochemical properties of the powder was evaluated, including particle size distribution and morphology analysis. Additionally, the impact on the dissolution rates of model drug ritonavir (RTV), chosen for its physicochemical characteristics as a poorly water-soluble drug belonging to the biopharmaceutical classification system class II,^15^ was also investigated. Excipients were selected based on compatibility with RTV^16–18^

## 2 Materials and Methods

### 2.1 Chemicals and Reagents

RTV (lot 04135, 97.6%) was kindly donated by Cristália Produtos Qumicos e Farmacêuticos Ltd. (Itapira, Brazil). Copovidone (COP, Plasdone S-630, average molecular weight of 47 kDa) was donated by Ashland Inc. (São Paulo, Brazil). Polyvinyl caprolactam-polyvinyl acetate-polyethylene glycol copolymer (SLP, Soluplus, average molecular weight of 118 kDa) was from BASF (Ludwigshafen, Germany). Purified soybean phosphatidylcholine (PPC, Lipoid S100, lot 579001160709) was from Lipoid GmbH (Ludwigshafen, Germany). Polyethylene glycol 400 (PEG) was obtained from Labsynth do Brasil (Diadema, Brazil). Colloidal silicon dioxide (CSD, Aerosil 200) was acquired from Sigma-Aldrich (São Paulo, Brazil). The solvents and other chemicals were analytical or HPLC grade.

### 2.2 HPLC Analysis

RTV quantification was performed by using a high-performance liquid chromatography (HPLC) device. The analyses were carried out in an Agilent 1260 Infinity II (Santa Clara, CA, USA) coupled with a ZORBAX Eclipse Plus C18 column (250 mm × 4.6 mm, 5 μm) kept at 25.0 ± 0.5 °C. The mobile phase consisted of acetonitrile and ultrapurified water (70:30, v/v) eluted in an isocratic flow at 1.0 mL/min. The injection volume was 20 μL, and the detector was set at 242 nm.^19^ The analytical method was validated following the International Conference on Harmonisation (ICH, 2024) guidelines for analytical procedures.^20^

### 2.3 Preparation of RTV Physical Mixtures and Extrudates

Before the thermal processing, physical mixtures (PMs) were prepared by mixing the copolymer (COP or SLP) with PPC (15 or 30%, w/w) by using a porcelain mortar and pestle. PEG 400 and CSD were added in formulations containing no PPC to improve the processability of the molten material within the hot melt extruder barrel. A summary of selected formulations is presented in Table 1.

**Table 1:** Ritonavir-Based Solid Dispersions of Copovidone and/or Soluplus with or without Phosphatidylcholine^a^

	**components (%, w/w)**					
**solid dispersions**	RTV	COP	SLP	PPC	PEG	CSD
COP-E	15	72			8	5
COP-P15	15	70		15		
COP-P30	15	55		30		
SLP-E	15		72		8	5
SLP-P15	15		70	15		
SLP-P30	15		55	30		

a PPC – soybean phosphatidylcholine, SLP – Soluplus, COP – copovidone, PEG - polyethylene glycol 400; CSD – colloidal silicon dioxide.

PMs were fed manually into a laboratory-scale vertical miniextruder (EHM 5 LabMaq, Ribeirão Preto, Brazil) equipped with a backflow channel for material recirculation. The extrusion barrel assembles a 16 mm twin screw (L/D = 16) with a helix angle of 1.97°. The extrusion was performed under a temperature gradient (130–140–150 °C) applied to 3 different heating zones from the top to the bottom. The material was kept under continuous recirculation for 3 min after feeding. The screw speed was set at 150 rpm, and the resulting torque was ≤ 10 N m. Finally, the extruded material was cooled to room temperature and milled in a cutting mill (Hamilton Beach, Southern Pines, NC, USA).

### 2.4 Thermal Analysis

DSC curves were obtained on a DSC-60 analyzer (Shimadzu, Kyoto, Japan). Samples of 4.0 ± 0.5 mg were placed in sealed aluminum pans and heated from 25 to 200 °C at a rate of 20 °C/min under a nitrogen atmosphere purged at 50 mL/min. The analyses were conducted for the raw materials, PMs, and milled extrudates (SDs) containing 15% (w/w) RTV.

### 2.5 XRPD Analysis

Phase identification and the crystallographic pattern of raw materials, PMs, and SDs were investigated in a Bruker D8 Discover Diffractometer (Madison, WI, USA). The monochromatic radiation was generated by a sealed tube containing a copper anode coupled to a Johansson monochromator for Kα1, operating at 40 kV and 40 mA, with Bragg–Brentano configuration θ to 2θ, from 4 to 50° in 2θ and a step of 0.01° in 2θ. The samples were rotated at 15 rpm during the measurement. The resulting analysis represents the arithmetic average of six individual measurements.

### 2.6 Particle Size Distribution and Morphology after Redispersion

PMs and their corresponding SDs were dispersed in ultrapure water (1:250, w/v) under magnetic stirring at 300 rpm for 30 min (IKA, Staufen, Germany). The resulting aqueous dispersions were vortexed for 5 min and centrifuged at 4000 rpm for 5 min (Solab Ltd., Piracicaba, Brazil). The supernatant was collected, and the particles’ mean size (d.nm) and polydispersity index (PdI) (*n* = 3) were analyzed in a ZetaSizer Nano-S (Malvern Ltd., Malvern, UK).

Photomicrographs were obtained in a transmission electron microscope (TEM) JEOL, model JEM-2100, equipped with energy dispersive X-ray spectroscopy (EDS) (ThermoScientific, Tokyo, Japan). Diluted samples were placed on a copper plate and kept there for 10 min. The excess was removed, and a drop of the uranyl acetate solution was added. The sample was dried at room temperature and analyzed at 200 kV.

### 2.7 In Vitro Dissolution Study

Nonsink dissolution tests were performed in a DT80 dissolution apparatus (Erweka, Heusenstamm, Germany) with a USP apparatus II operating at 50 rpm. The experimental condition was defined by the sink index equation (eq 1).

$$\mathrm{SI}=\frac{V\times C_{s}}{\mathrm{dose}}$$ 1

where *C*_s_ represents the equilibrium solubility of the crystalline drug, *V* is the volume of the dissolution medium (500 mL), and dose is the total amount of drug added to each vessel.^21^ Based on the experimental solubility assessment of the crystalline drug (0.004 mg/mL), the SI was set to 0.08 for the whole dissolution design.

Thereby, SDs and PMs were adjusted to an RTV mass equivalent of 25 mg. The dissolution medium consisted of purified water kept at 37 ± 0.5 °C, and aliquots of 2 mL were withdrawn at intervals of 5, 10, 15, 30, 45, 60, 90, and 120 min. Each sampling procedure was followed by the replenishment of fresh medium in order to keep the constant SI. The samples were centrifuged at 4000 rpm for 5 min (Solab Ltd., Piracicaba, Brazil), then 500 μL of the supernatant was diluted in acetonitrile and analyzed by UV–vis spectroscopy using a Genesys 50 spectrophotometer (ThermoScientific, MA, USA) at 242 nm. Dissolution of the RTV raw material was also evaluated under the same conditions. All assays were performed in triplicate.

Statistical analysis compared the percentual drug dissolved at 120 min using GraphPad Prism 7.00 Software (La Jolla, California, USA). Analysis of variance (ANOVA) followed by Tukey’s multiple comparison test was used to assess statistical differences (*p* < 0.05).

## 3 Results and Discussion

### 3.1 Drug Determination

RTV quantitation was performed using an HPLC method adapted from the study by Gandhi and Rasika.^19^ The RTV analytical curve ranged from 1 to 12 μg·ml^–1^ (*y* = 23.3119*x* + 2.372.7; *R* = 0.99), with *F* = 11.387 (test*-F*). The coefficient of variation values were lower than 5% for all concentrations and analysis levels. The limit of quantification was 0.22 μg·mL^–1^. Selectivity studies were conducted by analyzing the peak overlay in the chromatogram of RTV raw material and SD excipients. No interference peaks were observed in drug retention time (∼4.5 min), nor were any chromatographic properties compromised after mixing the drug and excipients (Supporting Information, S1).

The PMs and their corresponding SDs were thoroughly analyzed. The results demonstrated suitable chemical compatibility of the mixtures since the chromatograms evidenced adequate RTV recovery (101.4–105.8%) and the absence of further peaks (Supporting Information, S2).

### 3.2 Preparation of SDs by HME

Initial HME tests were conducted by using powdered mixtures comprising only COP (or SLP) and RTV, resulting in notably low yields. In order to enhance processability, PEG was introduced as a plasticizing agent in these mixtures. However, even with the addition of 5% (w/w) of this compound, the yield remained subpar (10%). CSD was also incorporated into the mixtures, enhancing processability (yield > 40%). The maximum improvement in the process yield was observed with 5% (w/w) CSD. In fact, this excipient facilitated the flow and promoted a better recirculation of the molten material inside the mini-extruder barrel. The resulting extrudates were characterized by a glassy and uniform aspect (Supporting Information, S3). Dispersions containing PPC exhibited a similar processability without needing additional adjuvants and showed a uniform opaque aspect (Supporting Information, S3). Indeed, the improvement in formulation processability caused by the coextrusion of polymer and lipid has already been reported.^22,23^ Next, six different formulations were prepared (Table 1) with sufficient recirculation time (3 min) since it is crucial to achieving a proper distributive mixing in mini-extruders;^13^ therefore, maximizing drug amorphization and drug-excipient interactions for all formulations.

### 3.3 Thermal and Diffractometric Behavior

Thermal transitions of the RTV raw material are presented in Figure 1. RTV exhibited a prominent endothermic event at 131 °C, which was attributed to drug melting (*T*_m_). The finding is consistent with previous literature report^24^ and indicates the crystalline nature of the RTV. Moreover, COP and SLP did not display endothermic events within the RTV melting range, whereas PEG and PPC exhibited endothermic events above 140 °C (Supporting Information, S4). The analysis shows that the thermal transitions of the named excipients do not interfere with RTV *T*_m_.

**Figure 1:**
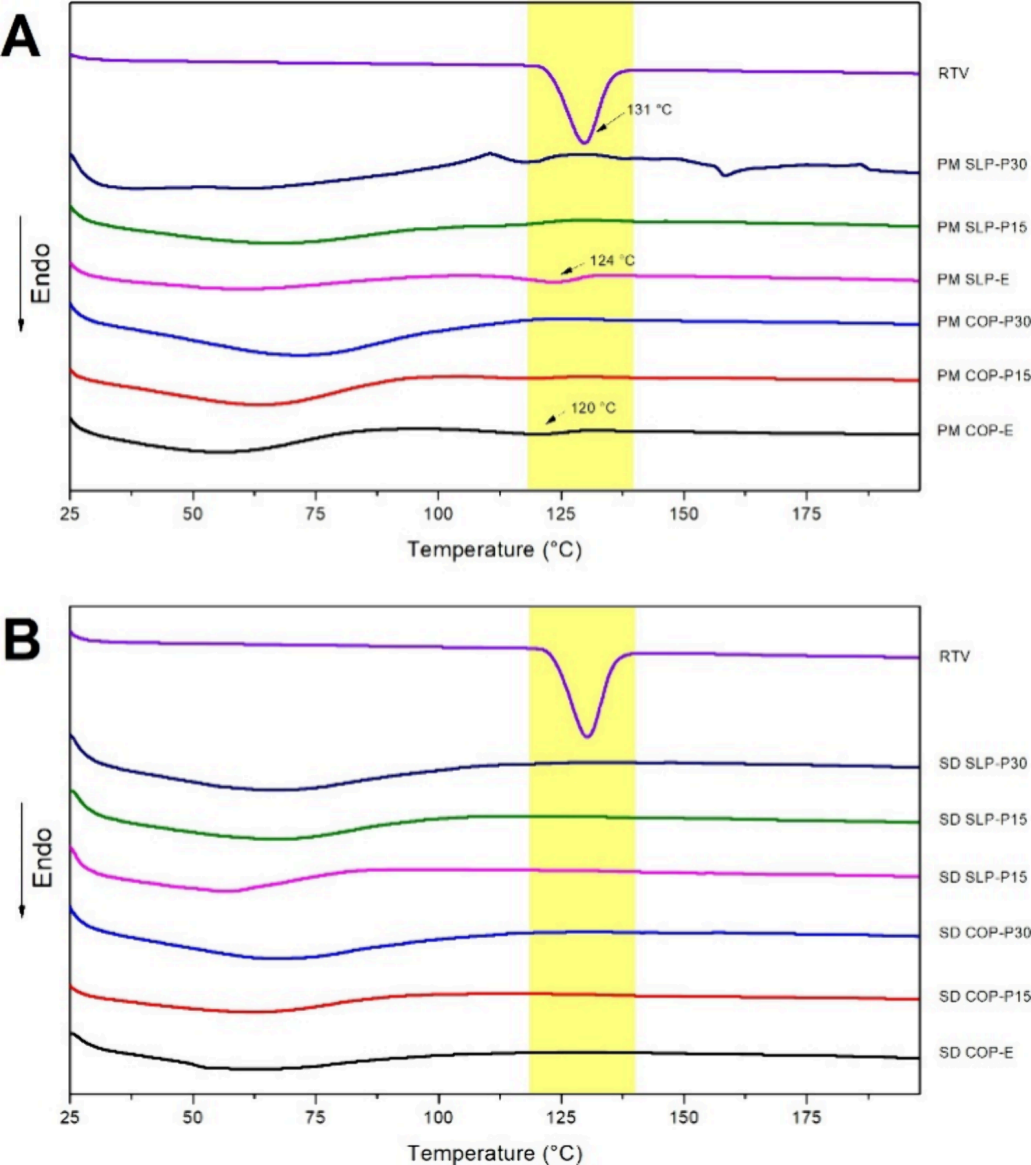
DSC curves of (A) physical mixtures (PM) and (B) solid dispersions (SD) containing 15% ritonavir (RTV, w/w) with up to 30% phosphatidylcholine (PPC, w/w).

The RTV melting (*T*_m_) in the mixtures was investigated by using the DSC curves of PMs (Figure 1A) and extrudates (SDs) (Figure 1B). The RTV *T*_m_ was shifted in COP and SLP PMs, but it could not be seen when PPC was added to the PMs. These results suggest notable drug solubilization by PPC during the heating step of the thermal analysis. Conversely, distinct RTV *T*_m_ was not identified in any of the extrudates. These observations indicate the drug’s complete amorphization/solubilization throughout the matrices via HME processing.

Corroborating the thermal analysis, XRPD revealed a pattern lacking long-range crystallographic ordering, confirming the successful conversion of RTV to an amorphous material on both binary and ternary extrudates. RTV isoform II presents the XRPD pattern with characteristic peaks at 9.51, 9.88, and 22.2° in 2Θ^25^ (Supporting Information, S5). In the PM diffractograms, the prominence of the same peaks indicates that the RTV crystallographic form II is conserved (Figure 2). At the same time, the characteristic halo associated with amorphous mixtures is evident in the SDs diffractograms. The XRPD patterns of the neat SLP, COP, and PPC are presented in Supporting Information (S6).

**Figure 2:**
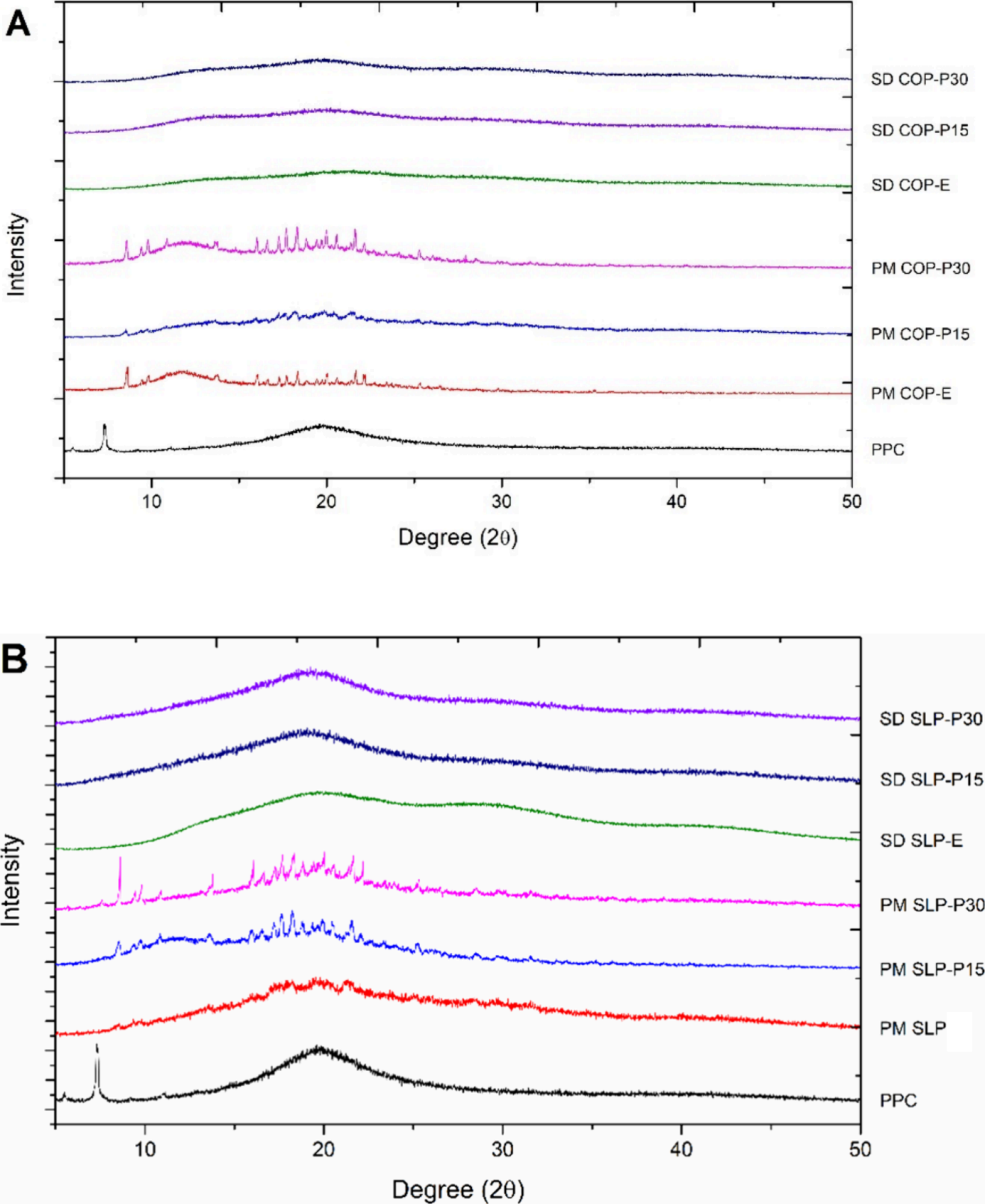
X-ray diffractograms of (A) physical mixtures (PM) and (B) solid dispersions (SD) containing 15% ritonavir (RTV, w/w) with up to 30% phosphatidylcholine (PPC, w/w).

### 3.4 Morphology and Composition Analysis

TEM micrographs of SDs containing COP evidenced the formation of aggregated particles (Figure 3A) following the morphology reported for COP particles in aqueous dispersions.^26^ DLS analysis indicated particles ranging from 80 to 300 nm, with a monomodal distribution (COP-E formulation). The mean diameter value was 172.9 nm with a PdI value of 0.111. The inclusion of PPC in COP formulations induced a noteworthy alteration in the particles’ morphological pattern, leading to a misshapen appearance (without aggregation) (Figure 3B,C) and a bimodal size distribution (Figure 3D). It was not possible to determine a reliable mean particle size or polydispersity index (PdI) from the COP and PPC mixtures. EDS analysis shows the presence of phosphorus in both small (Figure 3E) and large particles in the COP-P15 and COP-P30 formulations, suggesting the occurrence of polymer–lipid interaction in those structures.

**Figure 3:**
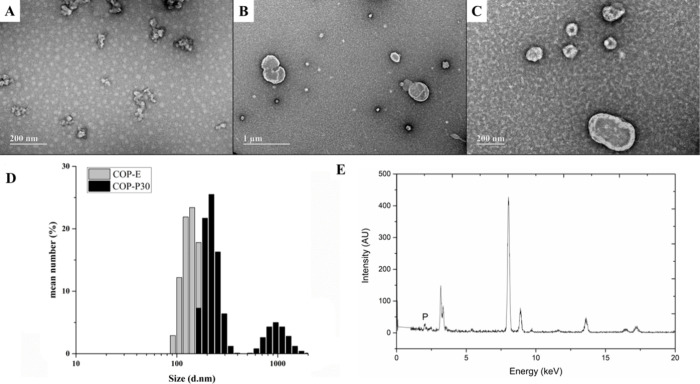
Photomicrograph of (A) ritonavir and copovidone solid dispersion (SD COP-E) and (B, C) ritonavir, copovidone, and 30% phosphatidylcholine solid dispersion (SD COP-P30). (D) Size distribution of copovidone-based solid dispersions (SD COP-E and SD COP-P30) and (E) energy-dispersive X-ray spectra of a small particle (∼100 nm) redispersed from SD COP-P30.

On the other hand, TEM images of SLP SDs show the presence of small uniform particles ascribed to polymeric SLP micelles (SLP-E) (Figure 4A).^26,27^ DLS shows particles ranging from 20 to 150 nm, with a mean size of 99.4 nm and a PdI value of 0.197. Upon PPC addition to the SLP SDs, larger particles emerged, suggesting the formation of multilamellar liposomal systems (Figure 4B,C). These structures were also seen in COP dispersions because PPCs are amphiphilic materials that spontaneously generate lipidic structures in an equilibrium state with exceeding water.^28^ Mean particle size and PdI value could not be reported for the SLP-PPC mixtures. The EDS analysis of small particles in SLP-PPC SDs revealed the absence of the lipid in such structures (Figure 4E), suggesting a lower degree of PPC- SLP interaction than that observed for COP formulations.

**Figure 4:**
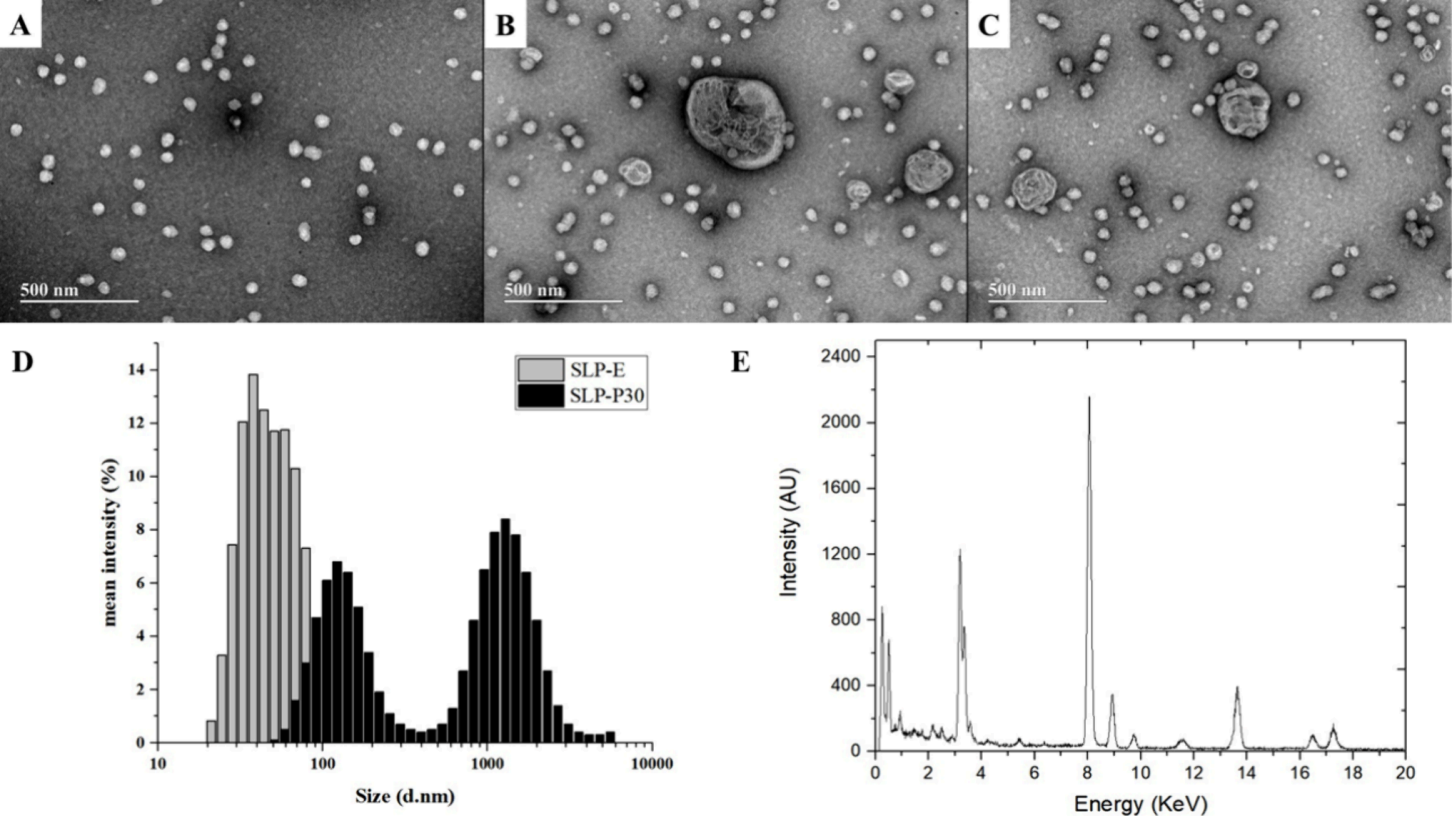
Photomicrograph of (A) ritonavir and Soluplus solid dispersion (SD SLP-E) and (B, C) ritonavir, Soluplus, and 30% phosphatidylcholine solid dispersion (SD SLP-P30). (D) Size distribution of Soluplus-based solid dispersions (SD SLP-E and SD SLP-P30) and (E) energy-dispersive X-ray spectra of a small particle (∼100 nm) redispersed from SD SLP-P30.

### 3.5 In Vitro Dissolution Studies

Figure 5 shows the RTV dissolution from PMs and SDs containing COP. The extrudates (SDs) exhibited a higher dissolution rate in all cases compared to PMs. These results were expected since the amorphous RTV harnesses a higher energetic state, reducing the thermodynamic barrier necessary to transition from solid to solution.^12,29^ Hence, it is desirable that HME processing also enables a supersaturating concentration of RTV in the dissolution medium, disclosing a potential drug delivery system for oral bioavailability enhancement.

**Figure 5:**
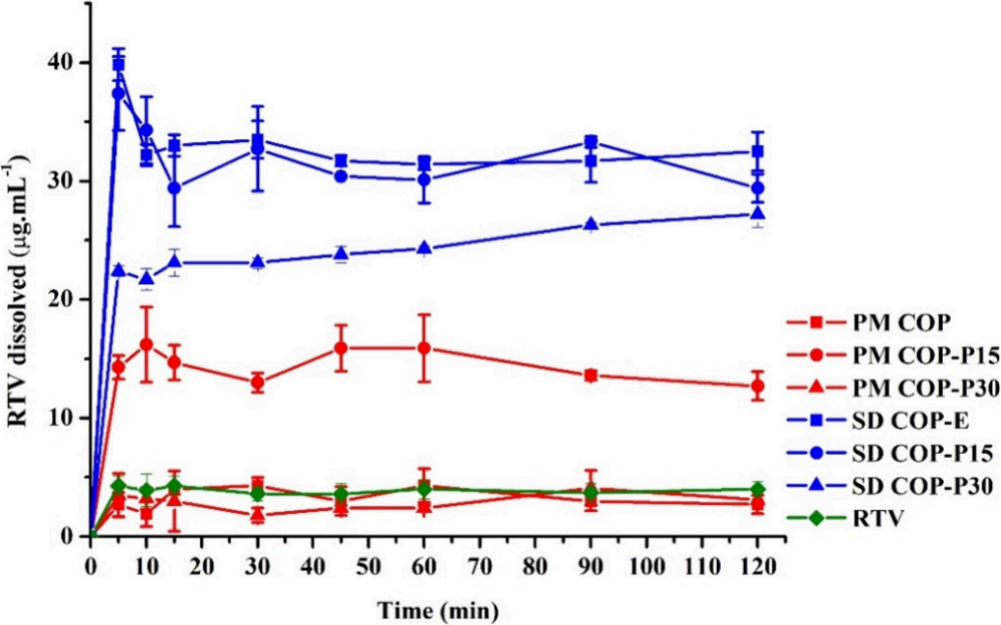
Dissolved ritonavir (RTV, μg·mL^–1^) from physical mixtures (PM) and solid dispersions (SD) based on copovidone, with increasing concentrations of phosphatidylcholine (PPC, 0–30%, w/w).

The COP-E showed a rapid RTV dissolution up to 40 μg/mL (10-fold higher than the solubility of the raw crystalline drug). The supersaturated state mediated by this copolymer is commonly attributed to drug interactions with the vinylpyrrolidone monomers of the polymeric chain, which are highly hydrophilic.^30^ The rapid achievement of the supersaturated state promoted by polymer-drug interaction at the molecular level was previously observed from the congruent release of RTV in COP SDs when the SD was prepared at drug loadings equal to or lower than 25%;^31^ however, a reduction of the dissolved concentration to 35 μg/mL is displayed at 10 min. Purohit and Taylor^32^ reported the RTV amorphous solubility in phosphate buffer, pH 6.8, as around 30 μg/mL. Despite the present study’s dissolution medium being purified water, it can be suggested that a liquid–liquid phase separation occurred in the initial stage of the dissolution assay until the RTV amorphous solubility was reached. In this case, the contribution of the drug-rich particles to solution “concentration” may not be captured due to their sedimentation in the centrifugation step, as suggested elsewhere.^33^

The addition of 15% PPC to the COP extrudate (COP-P15) did not lead to significant alterations in the RTV dissolution (*p* > 0.05) compared to COP-E. Thermal processing promoted a similar increase in the drug’s dissolution for both SDs, suggesting that the RTV is mainly associated with the polymer in the processed mixture. Another possible suggestion is that the RTV congruent release was not affected by the presence of PPC in the SD. The lack of PPC effect on RTV dissolution in this formulation is positive since it denotes that the phospholipid can be used as a plasticizer until 15% (w/w) concentration without compromising the performance of the SD. It is worth noting that upon a simple physical mixture, RTV seems not to be associated with the COP but is partially embedded in the lipid material. Such a suggestion is based on the RTV dissolution from PM COP-P15 and PM COP-E, which showed a notable improvement in RTV dissolution when 15% lipid was added to the mixture (4-fold). In summary, the COP-RTV interaction depends most on the thermal and mechanical stress during the HME.

Meanwhile, 30% PPC significantly reduced RTV dissolution in both PM and SD. We hypothesize that this limited dissolution might be associated with phospholipids’ effects on the drug dissolution mechanism from SDs. Notably, in PM containing 30% PPC, excess lipid material may have surrounded the RTV crystals, hindering drug dissolution by forming an insoluble covering.^34^ This finding suggests that the PPC concentration is a crucial factor in the RTV dissolution performance in the PPC—COP mixtures. Indeed, PPC positively affects processing yield in COP-based extrudates but should be used in concentrations equal to or lower than 15% (w/w).

The RTV solubilization in SLP micelles (SLP-E) (Figure 6) was less efficient in promoting the drug supersaturated state than that observed for COP SD (COP-E), which can be explained by a lower drug-polymer affinity and/or by the differences between RTV dissolution mechanisms from SLP and COP SD. Indeed, no investigation of the limit of congruency was reported for SLP-RTV SDs. It is relevant to note that drug-SLP interactions efficiently kept the RTV supersaturation during the dissolution experiment.

**Figure 6:**
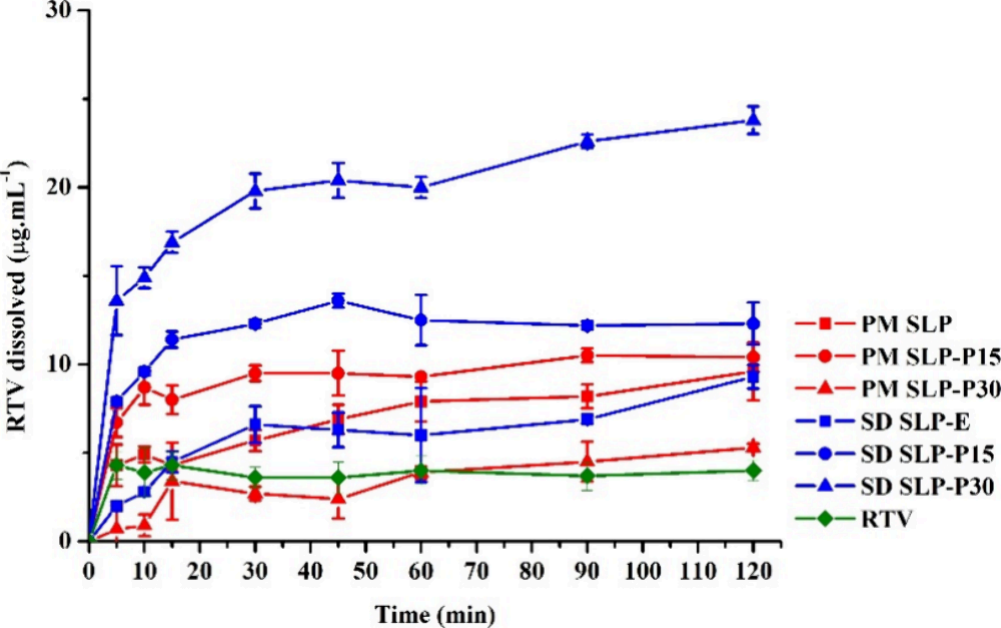
Dissolved ritonavir (RTV, μg·mL^–1^) from physical mixtures (PM) and solid dispersions (SD) based on Soluplus with increasing concentrations of phosphatidylcholine (PPC, 0–30%, w/w).

Conversely to what was seen for COP-PPC SDs, the addition of PPC in SLP extrudates led to a higher dissolved RTV concentration (*p* < 0.05) in a concentration-dependent manner. The lower RTV-SLP affinity suggested the relevance of adding a lipid surfactant to RTV-loaded SLP formulations. The possible effects of the PPC on the RTV dissolution mechanism from SLP particles can also be considered. Once again, it is noteworthy that the PPC-SLP SDs could maintain the RTV supersaturation state during at least 2 h of the experiment.

RTV from SLP PMs followed the same tendency observed for COP formulations. An intermediate PPC concentration in the mixture was able to improve drug dissolution compared with the SLP-RTV mixture prepared with no PPC; however, a higher PPC concentration probably hindered drug dissolution due to the formation of an insoluble barrier^34^

These findings indicate that the drug-polymer and drug-phospholipid affinity determines the relevance of adding a lipid in the polymer SDs; however, it seems clear that the lipid excipient is relevant in all cases to HME processability. Considering the RTV dissolution performance from SDs, PPC addition may be advantageous depending on the drug-polymer affinity.

## 4 Conclusions

This study aimed to determine the benefits of adding a phospholipid excipient in drug-polymer mixtures prepared by HME. As expected, the study showed the relevance of the thermal process in preparing amorphous SDs with superior dissolution performance. Relevantly, data showed that drug affinity with a particular polymer defines the role of the lipid in the formulation, and the polymer–lipid interaction did not influence the drug’s dissolution performance. It could be stated that adding phospholipids enables the thermal process without the addition of a low molecular weight plasticizer and a rheological agent. However, its role in improving drug dissolution depends on the drug-polymer affinity.

## Supplementary Material



## Notes

Chromatogram of ritonavir (RTV) and excipients (Figure S1); chromatogram of ritonavir extracted from the extrudates (Figure S2); extrudates obtained via hot-melt extrusion of ritonavir and copovidone (Figure S3); DSC curves of ritonavir and excipients (Figure S4); predictive X-ray powder diffractogram of Ritonavir isoform II (Figure S5); and X-ray diffractograms of neat Soluplus (SOL), phosphatidylcholine (PPC), and copovidone (COP) (Figure S6) (PDF)
