# Supplementary material for: Role of Phospholipids on Drug Dissolution in Polymer Solid Dispersions Prepared by Hot-Melt Extrusion
Source: ACS Omega. 2025 Jul 15;10(29):31501–8. doi: 10.1021/acsomega.5c01861 (PMC12311706; doi:10.1021/acsomega.5c01861)
Supplement: Supplementary file 1 [file ao5c01861_si_001.pdf]

Title: The role of phospholipids on drug dissolution in polymer solid dispersions prepared by hot-melt extrusion

Authors: Danilo Monteiro de Carvalho[1], Ana Carolina Mendes Lourenço[1], Guilherme Gomes Moreira[1], Fritz Eduardo Kasbaum[1], Ana Luiza Lima[2], Marcilio Cunha-Filho[2], Stephânia Fleury Taveira[1], Ricardo Neves Marreto[1,*]

[1] Laboratory of Nanosystems and Drug Delivery Devices (NanoSYS), School of Pharmacy, Universidade Federal de Goiás (UFG), 74605170, Goiânia, GO, Brazil.

[2] Laboratory of Food, Drug, and Cosmetics (LTMAC), School of Health Sciences, Universidade de Brasilia (UnB), 74910900, Brasília, DF, Brazil.

[*] **Corresponding author**: Prof. Ricardo Neves Marreto. E-mail: ricardomarreto@ufg.br. Universidade Federal de Goiás (UFG), Rua 240, Setor Leste Universitário, 74605-170, Goiânia, GO, Brazil. Phone: +55 62 3209-6037.

Supporting information, S1. Chromatogram of ritonavir (RTV) and excipients. The lack of analytical peaks in the drug's retention time indicates that the method is selective for detecting the analyte.

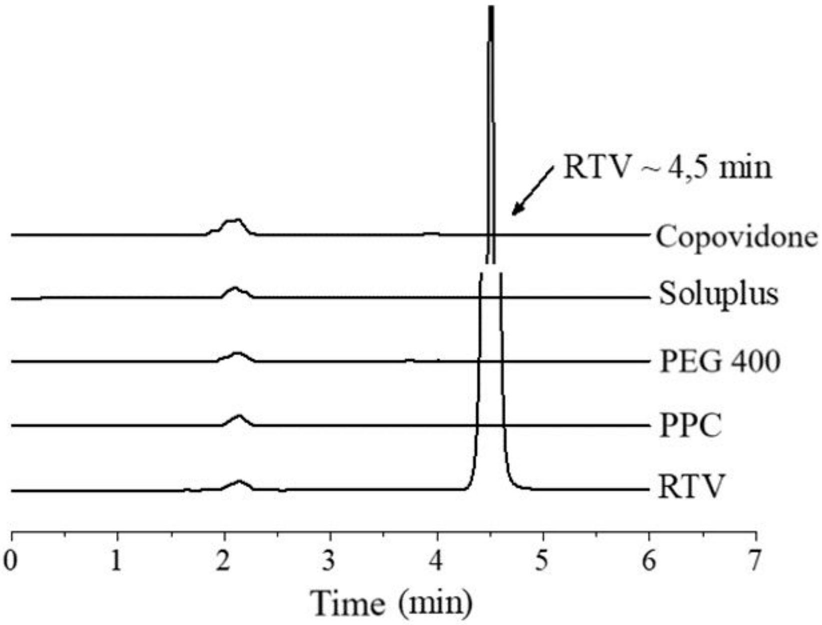

Supporting information, S2. Chromatogram of ritonavir extracted from the extrudates. The absence of scattered peak suggested that the material did not undergo significant degradation during processing

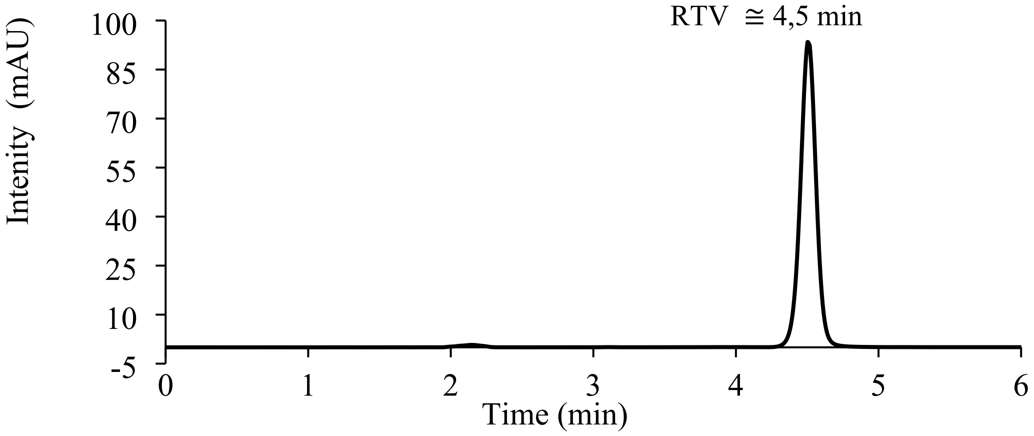

Supporting information, S3. Extrudates obtained via hot-melt extrusion of ritonavir and copovidone. (A) formulation without phosphatidylcholine (COP-E) and (B) formulation containing 15% (w/w) of phosphatidylcholine (COP-P15).

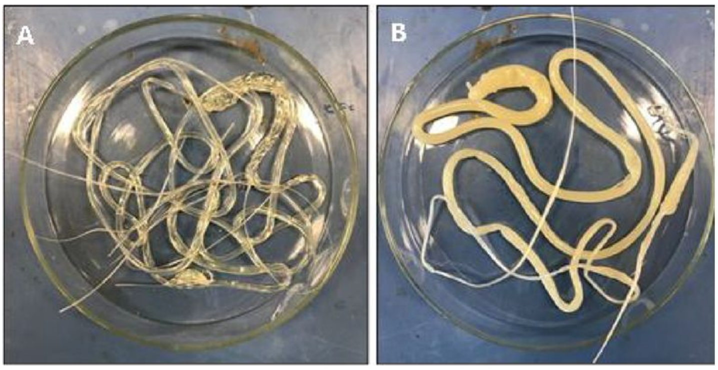

Supporting information, S4. DSC curves of ritonavir and excipients.

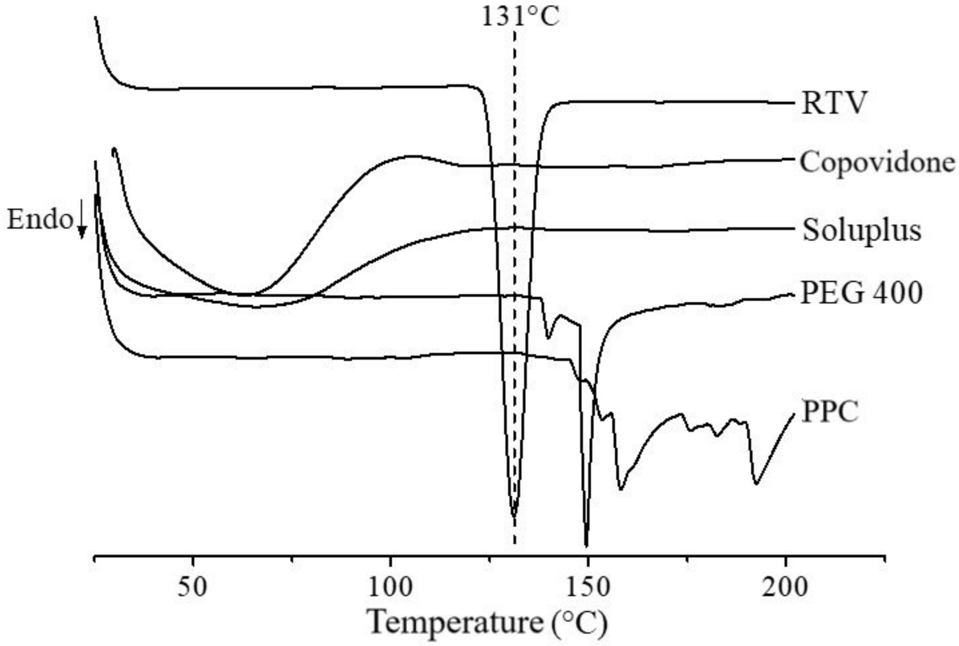

Supporting information, S5. Predictive X-ray powder diffractogram of Ritonavir isoform II. The data was calculated by Mercury 4.0, a crystallographic software for visualization, analysis, design and prediction.

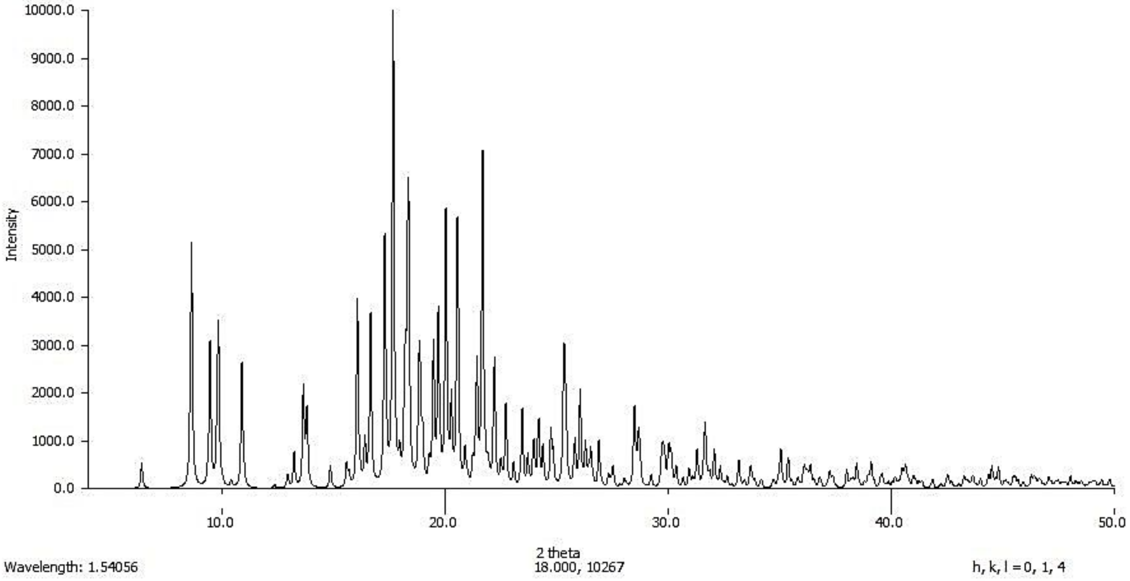

Supporting information, S6. X-ray diffractograms of neat Soluplus (SOL), phosphatidylcholine (PPC) and copovidone (COP).

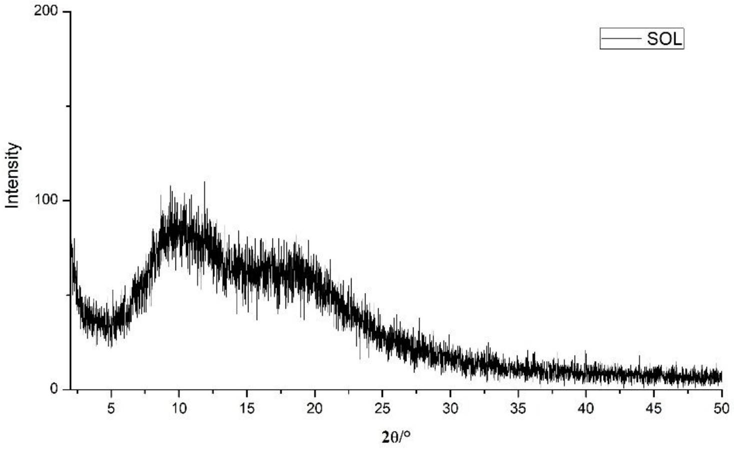

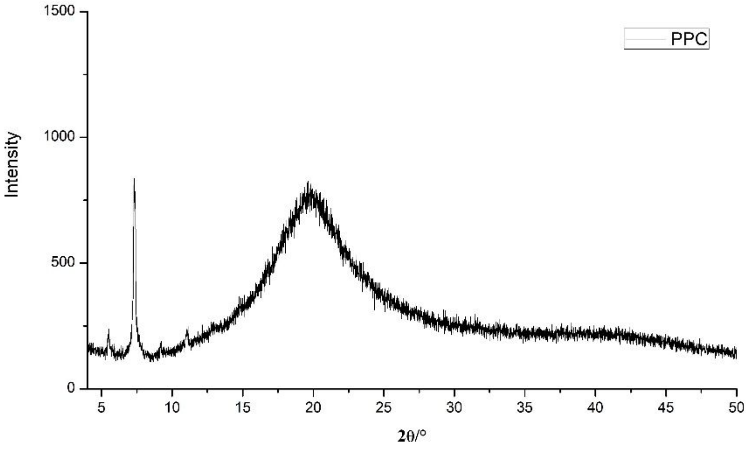

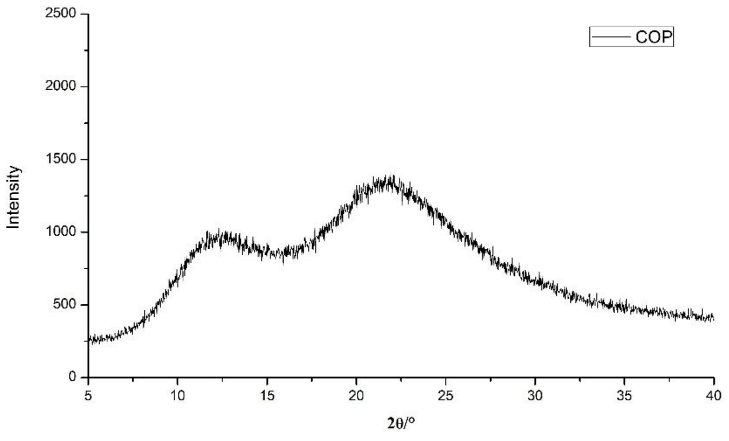
COP
Intensity
2500
2000
1500
1000
500
0
5
10
15
20
25
30
35
40
2θ/°
